# Youth Early-intervention Study (YES) – group interventions targeting social participation and physical well-being as an adjunct to treatment as usual: study protocol for a randomized controlled trial

**DOI:** 10.1186/s13063-015-0834-7

**Published:** 2015-08-05

**Authors:** Lillian Jean Gehue, Elizabeth Scott, Daniel Francis Hermens, Jan Scott, Ian Hickie

**Affiliations:** Clinical Research Unit, Brain and Mind Research Institute, The University of Sydney, Sydney, NSW Australia; Academic Psychiatry, Institute of Neuroscience, Newcastle University, Newcastle upon Tyne, UK

**Keywords:** Youth, Adolescents, Mental disorders, Physical health, Psychological intervention, Exercise, Functioning, Randomized, Cross-over

## Abstract

**Background:**

It is increasingly acknowledged that clinical interventions for young persons with mental disorders need to optimize social, vocational and physical functioning, and take into account developmental needs, rather than focusing only on the traditional target of psychiatric symptom change. However, few interventions for youth presenting to mental health services offer a coherent rationale for multi-faceted approaches that efficiently address all these targets.

This trial uses two facilitated group therapy modules (social and physical activity) as a vehicle for promoting clinical, cognitive, social and vocational change. The modules are an adjunct to usual treatments offered to youth attending mental health services in Sydney, Australia.

**Methods/Design:**

The design is a 2-arm, parallel group cross-over, randomized clinical trial (RCT) that examines the efficacy of this adjunctive youth early intervention program (called “YES”) for improving social, vocational, mental and physical health functioning in a trans-diagnostic sample of 120 young persons aged 14–25 years who are currently receiving a range of “usual treatments” for clinically diagnosed anxiety, affective and/or psychotic disorders.

Individuals who provide written informed consent are offered 2 group therapy modules (each comprising 4 hours per week for 8 weeks) with a 3-week “pause” between modules. Randomization determines whether individuals commence with module A or module B. The sample will be assessed pre-randomization, and at week 1 and week 8 (after completion of the first module), and at week 11 (commencement of second module) and week 19 (completion of second module). Final follow–up is 1-year post trial entry.

**Discussion:**

If the findings of this exploratory trial demonstrate benefits in the target domains, then it will be important to extend the research by undertaking: (a) a comparison of the YES program to a control intervention in a randomized controlled trial, (b) an explanatory study of putative mediators of change, and (c) a multi-center trial with a number of trained therapists offering the group modules combined with a longer follow-up period.

**Trial registration:**

Australian New Zealand Controlled Trial Registration: ACTRN1262400175673, Date: 16 July 2015

## Background

Adolescence and young adulthood are stages of life characterized by marked emotional, cognitive and physical developments [1]. In addition, the mid-teens to mid-twenties represent the peak age range for the onset of all adult-type major mental disorders [2]. However, the longitudinal trajectory of the presenting syndrome is not always predictable. For example, a depressive episode may herald the beginning of a lifetime of recurrent depression, but it may also be a precursor of a future bipolar disorder or of psychosis. Alternatively, the depression may represent an isolated episode of ill-health that never recurs [3]. Not only do the early stages of a mental disorder present a complex clinical picture, but also the symptoms of any underlying illness often co-exist alongside other psychological difficulties that are indicative of age-appropriate emotional/developmental problems (anxieties regarding, e.g. acceptance by their peer group, school performance, body image, etc.) [1, 4, 5].

The entanglement of mental health and emotional/developmental problems is further complicated by the emergence of physical health issues. It is increasingly clear that these youth may already have “risky behaviors” that are potentially harmful to future health and wellbeing (e.g. use of nicotine and alcohol, sedentary lifestyle, etc.), precursor syndromes for medical problems (metabolic abnormalities, etc.) and/or other evidence of vulnerability for the development of persistent physical disorders (cardiovascular disease, type 2 diabetes, etc.) that are likely to become burdensome in middle and older age [5]. In summary, mental disorders in youth may have an uncertain longitudinal course and may be complicated by emotional development difficulties and/or by co-morbid physical disorders that predict later multi-system problems.

There is a growing body of research that indicates that due to this complex interplay between emotional developmental difficulties and/or co-morbid mental disorders or physical illness, young people are at risk of increasing levels of social and occupational disability [6–11]. This decline in functioning is marked by poor educational outcomes, limited employment choices, and estrangement from family and personnel connections. As such, young people can become marginalized from their immediate as well as their extended communities, exacerbating their levels of impairment [6–8].

For many decades, interventions for young people with mental disorders mirrored the treatments offered to older adults, with the main goal being the reduction of the acute clinical symptoms and not necessarily addressing their social and occupational decline [7]. There have been gradual modifications to the format of the treatments to try to adapt them to meet the needs of a younger population, but it is only relatively recently that service-delivery changes have become more widely established (e.g. early intervention in psychosis: EIP) [12]. The introduction of EIP helped to widen the focus of interventions to include functional outcomes such as educational attainment and social participation; and the latest expansion of service options, with shifts towards youth-friendly mental health services [13], has now helped to increase awareness of the need to address emotional developmental issues. However, relatively few programs have attempted to augment these approaches with interventions that target physical well-being (e.g. sleep regulation, reduced nicotine, alcohol and/or substance use), physical activity (e.g. exercise programs), improving “metabolic health” (e.g. raising awareness of and using interventions that target diet and body weight, stabilize blood sugars) and/or social recovery (e.g. returning to a state of well-being) [5, 7, 13]. Given the risk of recurrence or persistence of mental and physical disorders from adolescence into adulthood, (cardiovascular morbidity, type 2 diabetes, etc.) [5], it is worthwhile to try to develop multi-faceted clinical interventions that are accessible to the majority of youth presenting to clinical services, and target recovery and physical well-being, as well as reducing psychiatric symptoms; especially if the approaches potentially have high benefit-to-risk characteristics [5].

This study will primarily examine the short-term efficacy of two modules that together form a youth-focused group intervention (called YES). The two modules are: Social Participation (module A), which focuses on social participation, recovery and interpersonal skills; and Physical Well-being (module B), which concentrates on behavioral activity and physical health. The YES intervention (i.e. modules A and B) is offered as an adjunct to the usual treatments that young people receive in clinical care. The study will comprise a randomized clinical trial (RCT) and will primarily assess functional recovery (changes in vocational status) associated with participation in each module (either A or B); and whether the order in which the modules are offered (module A followed by B, or module B followed by A) has any differential benefits for participants. Secondary outcomes to be assessed will include measures of clinical improvement, level of engagement with the modules, and a pilot evaluation of any changes in physical health status (e.g. including smoking, anthropometric, laboratory measured parameters such as cholesterol levels and metabolic outcomes).

## Methods

### Design

This is an un-blinded, two-arm, parallel group RCT with a cross-over design. The duration of the controlled phase of the RCT is 19 weeks. Participants will complete a pre-randomization assessment and after randomization to commence with module A or module B, they will complete further assessments at week1 and week8 (beginning and end of first module) and week11 and week19 (beginning and end of second module). The post-trial follow-up assessment will be undertaken 12 months after randomization.

The design will allow us to answer questions about the benefits of each module (comparison across groups between weeks 1–8; then comparison across weeks 11–19), and also whether benefits from or engagement with either module can be predicted by week 1 measures (e.g. are men more likely to engage with the module that targets activity whether this is offered for weeks 1–8 or for weeks 11–19? Do more individuals drop out in weeks 11–19 compared to weeks 1–8, no matter which module is being provided?).

### Setting, participants, recruitment and randomization

The study will take place at a youth mental health clinic linked to the Brain and Mind Research Institute (BMRI) at The University of Sydney, Australia. The participants will be young people who present to the mental health clinic, who complete a routine clinical “intake” assessment and the treating medical practitioner (usually a psychiatrist) confirms that they meet the eligibility criteria. The treating medical practitioner will then obtain written informed consent from the young person (plus consent of a parent or primary carer for those aged ≤ 16 years) to participate in the YES program.

The inclusion criteria are that the individual is: (i) aged 14–25 years; (ii) has a clinical diagnosis of an anxiety, unipolar, bipolar or psychotic disorder according to *Diagnostic and Statistical Manual of Mental Disorders, version IV* (DSM-IV) criteria [14]; (iii) is willing and able to give independent written informed consent to participate in the study (in addition, parental consent is required for those aged 14–16 years).

The exclusion criteria are that the individual has: (i) a clinically assessed IQ ≤ 70; (ii) a major neurological disorder, a medical illness which impacts on cognition, and/or a history of sustained head injury; (iii) inadequate English language skills to allow participation in groups (or completion of RCT assessment protocols); (iv) a current alcohol or substance misuse disorder and/or an acute psychotic or manic episode that impairs the individual’s ability to give informed consent and/or requires acute clinical treatment; (v) a risk of serious self-harm (as assessed by a medical professional); and/or (vi) participated in a research study of any structured psychological interventions within the preceding 12 months.

**Fig. 1:**
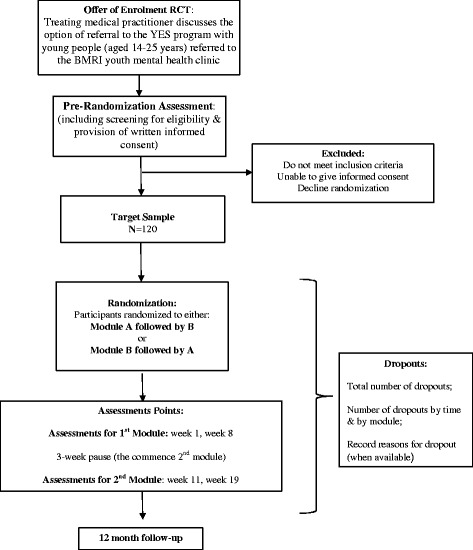
Recruitment, consent and randomization flowchart

Individuals who are eligible for the study will be randomized to start with either: module A (Social Participation) followed by module B (Physical Well-being) or to start with module B followed by module A. An independent person (i.e. a colleague not directly involved with the study) will undertake the randomization using a computerized random number generator program Fig. 1.

### Measures

There are some resource constraints for this study; hence for the pilot RCT, we will use the clinical assessment of the case (including the DSM-IV diagnosis) provided from the details recorded at intake by the treating medical practitioner. Outcome measures will rely mainly on self-report ratings of progress and outcome, but the treating practitioner, (who is blind to the randomization sequence) will provide an observer rating for the key primary outcome measure (of social and occupational functioning).

We selected ten self-report ratings and one observer-rating scale to measure outcomes associated with each target domain. These research assessment tools are widely used in mental health research and were selected partly because many of them are regarded as “standard” measures of outcomes in community, clinical and research populations and/or they have been found to have acceptable reliability and validity across age groups [5, 10–12]. Furthermore, the BMRI clinical services use many of these ratings routinely. As described in our previous publications, the clinic provides ongoing training and supervision in the use of clinical assessment tools and observer rating scales to ensure inter-rater reliability and consistent use [15].

Measures of outcome are divided into primary and secondary ratings, and the time points for these assessments are shown in Table 1.

**Table 1:** Schedule of enrollment, interventions and assessment time points

Phase	Pre-randomization		Post-randomization				Post-trial
Time point	Enrollment	Allocation	week 1	week 8	week 11	week 19	week 52 follow-up
Enrollment	x						
Clinical assessment (including DSM-IV diagnosis by treating medical practitioner)							
Eligibility screen	x						
Informed consent	x						
Randomization		x					
Allocates sequence of modules:							
AB or BA							
1^st^ Module							
2^nd^ Module							
Primary assessments:			x	x	x	x	x
Functioning - SOFAS and FAST							
Symptoms *-* SPHERE12			x	x	x	x	x
Disability *-* BDQ							
Self-esteem - RSEQ							
Vocational status:	x					x	x
Education, employment, receipt of benefits, etc.							
Engagement with intervention:				x		x	
Number of sessions attended							
Secondary assessments:			x	x	x	x	x
Levels of distress *-* K-10							
Social well-being *-* WHOQOL							
Physical health/Lifestyle:			x	x	x	x	x
Sleep profile - PSQI							
Exercise/Activity profile - IPAQ							
Tobacco and substance use *-* ASSIST							
Other measures:	x		x	x	x	x	x
Anthropometrics**:**							
BMI, body weight, waist circumference							
Metabolic screen and other blood tests:	x					x	x
Lipids, fasting glucose, FBC, LFTs, etc.							

*ASSIST* Alcohol, Smoking and Substance Involvement Screening Test, *BDQ* Brief Disability Questionnaire, *BMI* body mass index, *DSM-IV Diagnostic and Statistical Manual of Mental Disorders, version IV*, *FAST* Functional Assessment Short Tool, *FBC* full blood count, *IPAQ* International Physical Activity Questionnaire, *K-10*, Kessler Psychological Distress Scale, *LFTs* liver function test, *PSQI*, Pittsburgh Sleep Quality Index, *RSEQ* Rosenberg Self Esteem Questionnaire*, SOFAS* Social and Occupational Functioning Assessment Scale, *SPHERE 12* Somatic and Psychological Health Report, *WHOQOL* World Health Organization Quality of Life Scale

#### Primary outcome measures

i. Social and Occupational Functioning Assessment Scale (SOFAS): this is an observer-rated instrument that assesses functioning on a scale of 0–100, with lower scales suggesting more severe levels of impairment. The SOFAS is widely used in clinical research and practice and has been used in several research projects with young people that have been undertaken at the BMRI [5, 11, 16].
ii. Functional Assessment Short Test (FAST): this is a 24-item self-report measure that examines impairment across 6 domains: autonomy, occupational functioning, cognitive functioning, financial issues, interpersonal relationships and leisure time. It has been used in adults with bipolar disorder, schizophrenia and attention deficit hyperactivity disorder (ADHD) [17, 18]. It has been shown to have good psychometric characteristics with both a reliability and validity of about 0.9 each [18, 19]. This the first study to use the FAST in a population of 14–25 year-olds.
iii. Somatic and Psychological Health Report (SPHERE 12): is a 12-item self-report questionnaire which assesses 6 psychological (PSYCH-6) and 6 somatic (SOMA-6) symptoms. This screening tool has been shown to have a reliability of 0.9 a validity of 0.8 in populations aged 16 years and over [20, 21] and is widely used in youth mental health research [22].
iv. Brief Disability Questionnaire (BDQ): is an 8-item subjective rating of disability in everyday activities. It has been widely used in adult psychiatric populations [23, 24] and more recently has been used in youth populations [9, 11, 16].
v. Rosenberg Self Esteem Questionnaire (RSEQ): is a 10-item self-rated scale designed specifically for use in adolescent populations and has a reliability of 0.9 and a validity of 0.8 [25, 26]. A score representing a global measure of self-worth and for negative and positive self-esteem can be derived.

#### Secondary outcome measures

i. Kessler Psychological Distress Scale (K-10): this is a 10-item version of the Kessler scale that was developed for use in the US National Health Survey Interview (NHIS) and has been employed extensively around the world in (including Australia) across broad age groups in clinical and community settings. The K-10 gives a global measure of distress based on anxiety and depressive symptoms over a 4-week period, with a reliability of 0.9 and a validity of 0.8 [27, 28].

ii. World Health Organization Quality of Life Scale (WHOQOL): this 26-item self-rating gives a summary measure of functioning and social well-being across 4 domains: physical, psychological, social and environmental. It has been used across a wide range of age groups (from 12 to 97 years) and shows very high reliability and validity [29].

iii. Pittsburgh Sleep Quality Index: is a 24-item self-rating that gives a global measure of sleep quality. It assesses seven domains: sleep latency; sleep duration, habitual sleep efficiency, and sleep disturbances, use of sleep medication and daytime dysfunction. In young adult populations (aged 18–32 years) it has been shown to have good psychometric properties with both a reliability and a validity of about 0.8 [30, 31].

iv. International Physical Activity Questionnaire (IPAQ): is a 9-item questionnaire that assesses time spent walking, vigorous and moderate-intense activity, and sedentary activity. The IPAQ has been tested across populations and broad age groups in 12 countries and demonstrates a reliability of 0.8 and validity of 0.6 [32, 33].

v. Alcohol, Smoking and Substance Involvement Screening Test (ASSIST): is a 21-item tool that is used to self-rate current and past use of tobacco, alcohol and other substances. Its reliability is high (0.90) and it has been used worldwide, frequently in youth populations [34, 35].

Other Measures: anthropometric measurements (body mass index (BMI), waist circumference, etc.) and blood for pathology tests (e.g. metabolic and inflammatory markers) are collected routinely at the youth mental health clinic at the BMRI. These routine data and results of tests are reviewed by the treating medical practitioner and repeated as deemed necessary throughout the course of any treatment program [5, 15]. For this study we have added a specific schedule for treating medical staff to repeat these measures to fit with key assessment points in the RCT (see Table 1).

The routine intake assessment also collects data on educational or vocational status, and receipt of benefits. Again we will specifically ask clinicians to record this at selected time points throughout the course of the study.

For the purposes of the study, we will have access to records giving basic details of medication and/or other treatments received and will review these at the beginning of the study, at the end of the YES program, and again at the final follow-up. (However, this information will not be included in the formal analysis as resource constraints prevent us from collating information in sufficient detail to be able to interpret if findings are significant, e.g. differences dosages of medication prescribed, week-by-week medication changes, or levels of adherence, etc.)

### YES intervention

A particular challenge for delivering a psychological intervention in youth is to find non-threatening ways to engage individuals who exhibit different levels of self-confidence and/or vary in their actual levels of social and/or inter-personal skills [10, 11]. Hence, specific behavioral or psychological techniques may be less efficacious if the attendees feel too anxious about their performance on tasks (such as concentration exercises or changing behaviors) to engage in the process and such issues may lead to premature drop-out if the individual begins to feel stressed by participation in the group program. However, if individuals are enabled to overcome these potential barriers, the group format can offer peer support and be a suitable vehicle for delivering a wide range of technologies that increase rather than decrease the chance that the individual will achieve the goals they have set for themselves.

Both the “social” and “physical” modules incorporated in the group intervention are of 8 weeks duration and there are 2 sessions a week that together provide about 4 hours of group work (1 session is slightly longer than the other). This translates as 16 sessions (32 hours) per module – which is in keeping with many complex therapies offered to adults that target a specific major mental disorder (such as cognitive behavioral therapy (CBT) for schizophrenia, group psycho-education for bipolar disorder, and is longer than the interpersonal therapy (IPT) intervention for depression). It is our view that the duration is justified as there are multiple targets for this program, (including mental health, emotional/developmental issues and physical health) and that at this phase of their personal development many young people benefit from additional time to allow them to engage with the therapy and get used to attending the group and then benefit further from repetition of the key learning points through a range of different cognitive, behavioral and social mechanisms and interactive exercises and experiences.

To help foster familiarity with the format of the program, the structure of the session remains relatively fixed throughout the course of each module. However, the content varies from week to week and between modules. A range of different formats and modalities (art work, Internet programs, You-tube or similar open-access videos, phone “apps,” brief interactive group games or exercises) are used to ensure there are opportunities to engage youth with different skills, abilities and preferences and to anchor the learning exercises, (such as cognitive remediation components) to real-world situations.

A trained professional with a specific knowledge and skills facilitates the group on each topic/subject area. For example, “Anxious Art” (Social Participation: module A) is facilitated by an art teacher at the National Art School of Australia, whilst cardio boxing (Physical Well-being: module B) is co-ordinated by an accredited fitness instructor. The maximum group size is 15 young people starting each module (either A or B). Allowing for the small anticipated drop-out rate (estimated from our prior experience), we predict 12-regular attendees per module session. A pilot manual of topics and program content, including resources and teaching aides required, will be updated after the trial and made available.

Feedback by group participants and discussion of learning points with the group leader are often delivered during less formal parts of the session such as during an “afternoon tea and cake” break.

#### Synopsis of program content

This ranges from group activities focusing on executive skills (concentration, attention, planning), cognitive-emotional regulation (use of Mindfulness, managing rumination and mood swings), through to reducing health risk behaviors (nicotine and alcohol consumption) and enhancing health-promoting behaviors (periods of moderate-intensity to high-intensity exercise).

#### Extended session (2.5 hours)

When participants arrive, they are welcomed and given a quick overview of the session program (see Table 2). The “topic of the day” (e.g. mood regulation, problem solving, etc.) is briefly outlined and any experiential tasks are explained, written or other resources are distributed and the structured intervention begins (e.g. tennis (Physical Well-being Module) or Anxious Art (Social Participation Module). Next, there is a 25–30 minute break when participants listen to a “tea-cup talk.” This is a brief, informal lecture on a specific topic followed by an interactive discussion, summarizing and feedback. Useful webpages and phone “apps” are also identified. At the completion of the talk, participants return to the structured intervention activity for about 1 hour. To signal the close of the session, “housekeeping issues” (dates of specific activities, equipment needed, etc.) are addressed. Participants are thanked for attending and encouraged to attend the next session.

**Table 2:** Format of extended group session (2.5 hours)

Activity	Duration
Welcome and overview	5 minutes
Introduction of topic	5 minutes
Group activity	60 minutes
Module A- example: Anxious Art	
Module B- example: tennis	
Re-introduce topic, distribute hand-outs	5 minutes
Activity component	5–10 minutes
Skills component	5–10 minutes
Summary and feedback	5 minutes
Return to group activity (Continue module A or module B activity)	50 minutes
Reminders and close of group	5–10 minutes

#### Short session (1.5 hours)

When participants arrive, they are welcomed and given an overview of what the session will include. Any housekeeping issues (dates, equipment use, etc.) are addressed. A short talk is given on a specific topic (5–10 minutes) and hand-outs are distributed. This is followed by a 1-hour activity such as cardio boxing (module B: Physical Well-being) or e-Health Lounge (module A: Social Participation). The session concludes with a reminder about the session topic of discussion. Participants are then thanked and encouraged to attend the following week.

### Sample size/Power calculation

This feasibility study will allow us to undertake sample size calculations for future multi-center studies and inform the selection of outcome measures. There are no studies that have undertaken group interventions that target the same variables and outcomes specifically as this study and, most importantly, as both modules are deemed “active” interventions, we do not anticipate large between group differences associated with the modules. However, based on previous data from published clinical observational studies and controlled trials (that have estimated means scores on the SOFAS and FAST to be about 60–70 and 20–25 at baseline and clinically significant improvement to be associated with score changes of about 15–25 % over about 10–20 weeks), we have made a conservative estimate that each module can produce a statistically significant improvement in the scores (*p* < 0.05 with > 80 % power) on the 2 most important primary assessments: namely, the continuous measures of functioning (the FAST and SOFAS) of > 5 points between week 1 and week 9, and between week 11 and week 19 (we have not made any predictions associated to the 12-month follow-up).

Randomization of 120 individuals, with predicted retention of about 90–100 individuals at week 8 will give a sample size that is sufficient to detect clinically significant changes in functioning with medium to large effect sizes. Also, we predict this initial recruitment level would provide follow-up assessments on about 72–80 individuals at week 19 and about 66–70 individuals at 12 months.

Information on categorical outcomes (such as vocational status) is likely to be available on a larger sample, as it does not require the individual to participant in self-rated or observer-rated assessments, nor attendance for laboratory tests. Feedback is encouraged by all participants and is included as part of the week-to-week “summary/discussion” at the end of each group session. As such, whilst this information will be used for planning modifications to the programme, it is not being formally collated and analyzed.

### Statistical analysis plan

All data are entered into and analyzed descriptively using SPSS, version 21 (SPSS Inc., Chicago, IL, USA). As this research was conducted in health service settings (with participants, clinicians and/or administration information being utilized\, there are variable rates of missing data, which will be taken into account in the analysis plan.

Primary analyses will be based on the intention-to-treat (ITT) sample: i.e. everyone who was randomized will be included in the analysis regardless of participation (full or partial or none) in the intervention groups. The primary outcome is the change in FAST (self-rated) and SOFAS (observer-rated) score pre-intervention to post intervention (e.g. between weeks 1–8 and then weeks 11–19). A preliminary analysis will be reported on week-8 outcomes once the target sample has completed this stage.

Further analyses will evaluate the scores at the end of the follow-up period and changes in secondary outcome measures. Categorical outcomes, such as vocational status will be examined using simple (chi-squared) and logistic regression models.

Mixed-model repeated measures analyses may be used for some analyses because of the ability of this approach to include participants with missing data without using discredited techniques such as last observation carried forward.

Additional analyses will include per-protocol analyses and statistical methods to explore factors that moderate outcomes, including attrition rates and, if appropriate, levels of presenting severity associated with significant social, clinical or functional improvement or change in vocational status.

Finally, some basic analyses will be undertaken of anthropometric and metabolic screening data. The latter will include a review of the uptake of these screening tests by study participants, and any barriers to this approach, as well as analysis of the findings. This pilot data will be used to inform power calculations for future studies.

### Ethical considerations

The Human Ethics Research (HREC) Committee of the University of Sydney Australia has approved this study (2012/1639).

The group interventions are designed as an adjunct, not an alternative to usual treatments offered by the youth mental health services. As such, all participants are encouraged to continue to follow the healthcare advice of their treating clinicians and to remain in their care, as well as attending the groups. This usual treatment may include medication, counseling and/or referrals to a range of specialist mental health treatments or services.

Individuals who decline to complete follow-up questionnaires or assessments will be allowed to complete any groups they wish to attend. Also, in the follow-up phase (which includes the third school term), individuals may be allowed re-entry into groups if this is deemed clinically appropriate. For these cases, the data on weeks 1–19 will be included in the ITT analysis, but they will be excluded from some per-protocol analyses.

## Discussion

This trial examines the YES program which comprises two group modules that address mental health, developmental and physical health issues that are common features of the clinical presentations seen in young people attending youth mental health services. The modules use a youth-friendly format to try to maximize engagement by men and women. If the findings of this exploratory trial demonstrate benefits in the target domains, then it will be important to extend the research (a) to compare the YES to a control intervention in an RCT; (b) to undertake an explanatory study of putative mediators of change; and (c) to conduct a multi-center trial with a number of trained therapists offering the group modules and to examine outcomes over a longer follow-up period.

### Trial status

Recruitment is underway, and a poster describing the study was presented at the meeting of the International Society of Affective Disorders, Berlin, April 2014.

## Abbreviations

ADHD: attention deficit hyperactivity disorder
ASSIST: Alcohol, Smoking & Substance Involvement Screening Test
BDQ: Brief Disability Questionnaire
BMI: Body Mass Index
BMRI: Brain & Mind Research Institute
CBT: Cognitive Behavioural Therapy
DSM-IV: *Diagnostic and Statistical Manual of Mental Disorders, version IV*
EIP: early intervention in psychosis
FAST: Functional Assessment Short Tool
FBC: Full Blood Count
HREC: Human Research Ethics Committee
IPAQ: International Physical Activity Questionnaire
ITT: Intention to Treat
IPT: Interpersonal Therapy
K-10: Kessler Psychological Distress Scale
LFTs: Liver Function Test
PSQI: Pittsburgh Sleep Quality Index
RCT: Randomized Clinical Trial
RSEQ: Rosenberg Self Esteem Questionnaire
SD: Standard Deviation
SOFAS: Social and Occupational Functioning Assessment Scale
SPHERE 12: Somatic and Psychological Health Report
WHOQOL: World Health Organization Quality of Life Scale
YES: Youth Early-intervention Study (program)
